# Modeling schistosomiasis spatial risk dynamics over time in Rwanda using zero-inflated Poisson regression

**DOI:** 10.1038/s41598-020-76288-8

**Published:** 2020-11-06

**Authors:** Elias Nyandwi, Frank Badu Osei, Tom Veldkamp, Sherif Amer

**Affiliations:** 1 Faculty of Geo-Information Science and Earth Observation, University of Twente, Enschede, The Netherlands; 2 Geographic Information Systems and Remote Sensing Centre, University of Rwanda, Kigali, Rwanda

**Keywords:** Environmental sciences, Infectious diseases

## Abstract

The recorded clinical cases of *S. mansoni* at primary health facility level contain an excessive number of zero records. This could mean that no *S. mansoni* infection occurred (a true zero) in the health facility service area but it could also that at least one infection occurred but none were reported or diagnosed (a false zero). Standard statistical analysis, using exploratory or confirmatory spatial regression, fail to account for this type of data insufficiency. This study developed a zero-inflated Poisson model to explore the spatiotemporal variation in schistosomiasis risk at a fine spatial scale. We used environmental data generated at primary health facility service area level as explanatory variables affecting transmission risk. Identified risk factors were subsequently used to project the spatial variability of *S. mansoni* infection risk for 2050. The zero-inflated Poisson model shows a considerable increase of relative risk of the schistosomiasis over one decade. Furthermore, the changes between the risk in 2009 and forecasted risk by 2050 indicated both persistent and emerging areas with high relative risk of schistosomiasis infection. The risk of schistosomiasis transmission is 69%, 29%, and 50% higher in areas with rice cultivation, proximity to rice farms, and proximity to a water body respectively. The prediction and forecasting maps provide a valuable tool for monitoring schistosomiasis risk in Rwanda and planning future disease control initiatives in wetland ecosystem development context.

## Introduction

Schistosomiasis is an acute and chronic parasitic disease which is widespread in many regions in the Global South^1^. People become infected during agricultural, domestic, occupational and recreational activities which expose them to infect and get infested from water^2^. In Rwanda, *Schistosomiasis mansoni* (*S. mansoni*) infection also constitutes a significant public health problem. It has an overall country prevalence of 2.7%, which is an average from minimum value of 0.0% for most of mapping units to as much as 69.5% among school children^3^. The nationwide prevalence surveys of 2007–2008 and 2014 also tested for Schistosomiasis haematobium via urine samples but did not find positive cases implying that only *Schistosomiasis mansoni* is endemic in Rwanda. In response, the Neglected Tropical Disease (NTD) control program, was established in 2007. The NTD control initiative has developed capacity in disease diagnosis and treatment, and established Mass Drug Administration (MDA) campaigns in endemic areas. Furthermore, sentinel sites (12 schools and 2 villages) were selected based on nationwide prevalence mapping, for schistosomiasis surveillance^4^. However, high rates of infection in traditionally endemic areas are persisting^5^ and new *S. mansoni* foci within previously non-endemic zones are appearing^6,7^.

Prior research in other countries in the Global South demonstrated that increased *S. mansoni* transmission can be associated with the conversion of wetlands for intensified agricultural production, irrigation schemes and construction of dams^8^. In Rwanda, wetland conversion is one of the major mechanisms to increase (irrigated) agricultural production, and so ensure food security for the growing population^9^. Climatic factors (temperature and rainfall) can also influence disease transmission^10,11^. Climatic factors also have a significant, and geographically varying, impact on the size and spatial distribution of Rwandan wetlands^12^.

In an earlier study, we investigated *S. mansoni* transmission in Rwanda using routinely collected data of confirmed cases recorded at primary health facility level^13^. Confirmed case data were aggregated to geographically bounded health facility service areas (HFSAs), and merged with population data to generate incidence rates. The analysis demonstrated that routinely collected health records can be used to capture the spatial and temporal dynamics of *S. mansoni* transmission at a fine-grained spatial resolution*.* Moreover, in our study socio-economic risk factors were excluded from the explanatory risk factors (including access to improved water for domestic uses, proper sanitation, wearing shoes, bush defecation behavior, etc.)^14^. Those factors are represented by qualitative (%ge) and not spatially explicit figures—result of integrated living conditions surveys extrapolated at district level (10 times + bigger than a HFSA). It is assumed that the missing information on human ecology related to rice cropping landscape, such as putting-off shoes while cultivating, increased behavior of defecating in the bush and use of river/wetland water (while far from their home toilet and other facilities) is captured in significant relation of infection (new hotspots) and distribution and trend of wetland and rice cropping (as proxy).

However, a considerable number of HFSAs have zero recorded clinical cases of *S. mansoni.* A HFSA where no cases were recorded could indeed be an HFSA where no *S. mansoni* infection occurred (a true zero) or it could also be an HFSA where at least one infection occurred but none were reported or diagnosed (a false zero). Standard statistical analyses, such as exploratory or confirmatory spatial regression, fail to properly account for such data insufficiency and can therefore not appropriately estimate infection risk for areas with zero confirmed cases^15,16^. Failing to account for this can lead to unstable estimations of infection patterns. Spatial zero-inflated Poisson methods can handle this type of data problems and are increasingly used more recently for disease risk modeling^17^ and forecasting^1^.

To date, however, such an analysis has not been done in Rwanda. We adopt a two-staged approach. The *first* stage of the analysis identifies the (available and key) risk factors associated with *S. mansoni* transmission. The *second* stage presents a forecast of the expected future spatial pattern of *S. mansoni* transmission for the year 2050. The zero-inflated Poisson model developed to forecast the 2050 situation incorporates the planned expansion of rice cultivation in wetlands as well as the anticipated climate-induced changes in rainfall and air temperature in Rwanda. The approach developed also has application potential for spatial analysis and modeling of other environmental diseases in Rwanda and in other geographic settings.

## Methods

### Study area

Rwanda, a small, densely populated, landlocked country of 26,338 km^2^ in the Great Lakes region of central-eastern Africa, is administratively divided into four provinces and City of Kigali, 30 districts and 416 sectors^16^ and has a population of about 12. 5million (2018). The majority of the population is engaged in agriculture (85%). Rwanda has a relatively dense and dynamic hydrological network with many lakes and rivers and numerous floodplains and wetlands covering around 10% of the land surface^12^. In the framework of Vision 2020 and the Economic Development and Poverty Reduction Strategy (EDPRS), rural development and agricultural transformation are spearheads for rapid and sustainable development^17,18^. Wetland conversion is one of the major mechanisms to increase agricultural production, ensure food security for the growing population, and achieve Sustainable Development Goal 2.

### Parasitological data

The anonymized confirmed cases of *S. mansoni*, recorded per primary health facility, were aggregated at HFSA level and provided by the Malaria and Other Parasitic Diseases Division of the Rwanda Biomedical Centre (RBC/M&OPD). The dataset consists of laboratory-confirmed cases from all public and faith-based health facilities for the period January 2001 to December 2009. The RBC/M&OPD collects stool specimen from suspected patients and test using Kato-Katz techniques. At least two slides per stool specimen are always prepared and read by two microscopists. Rwandan health facilities are well-equipped and have adequately trained laboratory technicians to accurately conduct laboratory testing^4^.

We acknowledge that incidence rates based upon routinely collected health records, as used in this study, do not fully substitute prevalence data but argue that these rates, in the case of Rwanda, represent a reliable proxy of the spatial, and spatiotemporal distribution of *S. mansoni* infection. Nyandwi et al.^13^ motivated that patients with clinical symptoms of schistosomiasis will generally seek medical care in a nearby health facility because of high levels of geographic access, absence of affordability issues given widespread community-based health insurance, non-existence of traditional medicine for schistosomiasis, and a well-developed network of community health workers. Nyandwi et al.^13^ also found a strong correlation (R^2^ = 0.79) between location–specific prevalence data recorded at sentinel sites throughout the country and incidence rates of schistosomiasis in corresponding HFSAs.

Although routine health data does not capture all infections, but the systematically recorded incidence at small scale largely reflect the spatial pattern of infection or of risky zones. Furthermore, that specific fraction is well captured by the Rwandan HMIS data considering that: (1) *S. mansoni* infection is diagnosed via microscopic identification of eggs in stool samples in the laboratory of the health facility; (2) Physical accessibility to health Centre is not problematic, with vision 2020 envisaged and achieved target, allowed to have a health facility within a walking distance of at most 5 km to the nearest health facility; (3) the increased financial accessibility and behavior for health service seeking through large proportion of adhesion to Community Based Health Insurance as the patient pay only 10% of the total cost of service and medication; (4) the committed community health workers are actively stimulating patient to visit the health facility and reporting to the supervisors any suspected health problems; (5) there is no traditional medicine used for schistosomiasis in Rwanda; (6) the country is known for being an ICT Hub of Africa, thus the routine data are systematically collected and reported at each health Centre using the web-based software platform (DHIS 2) by an IT professional who is entering their monthly health records into directly the national database and (7) the impact of creation of NTD control program and detection of new hotspot of schistosomiasis. The reported number of confirmed cases do not have the exact location of patient, they are aggregated at the smallest spatial scale (HFSA) since patient details are not shared, but the organization of Community Based Health Insurance Scheme until 2012 was assigning to the community a health facility for the first visit, without that you were supposed to pay fully. Survey has also proven that even in urban area the number of people seeking for health service from private institutions was less than 4%.

### Environmental risk variables

#### Associated environmental variables

**Figure 1:**
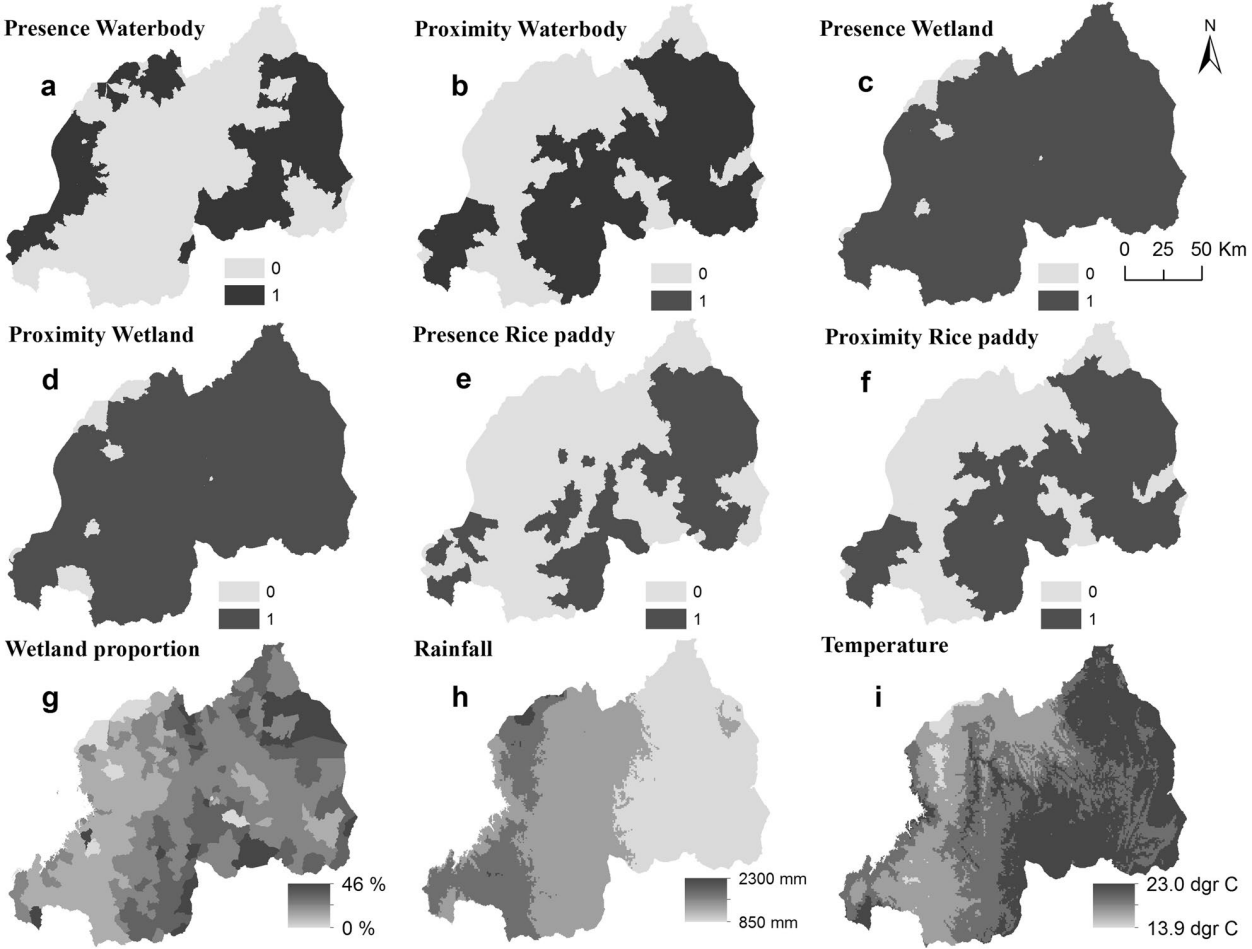
The frequency of HFSAs with/without confirmed cases of schistosomiasis.

The environmental variables used for modeling were extracted at the individual HSFA level as follows. Rice cultivation (*Rice*), proximity to rice cultivation (${d}_{Rice}$), and proximity to water bodies (*d*_*Water*_ ${\mathrm{d}}_{\mathrm{Water}}$) were modeled as categorical variables, whiles wetland proportion^19^ (*Wet*), rainfall (*Rain*) and air temperature (*Temp*.) were modeled as continuous variables. The variable *Rice* is an indicator of existing rice cultivation in an HFSA. The variables *d*_*Rice*_ and *d*_*Water*_ are HFSAs that have rice cultivation or water bodies within a 5 km distance. The cut-off value of 5 km is based on prior studies on *S. mansoni*^20,21^. Detailed descriptions of how these variables were extracted can be found in our previous study^12^. The categorical and continuous variables are illustrated in Fig. 1.

#### Projection of future spatial trend of risk factors

The second objective of the study was to forecast *S. mansoni* transmission risk. We address the following question: considering the future (2050) spatial configuration of risk factors, what would be the expected spatial distribution of *S. mansoni* risk? In order to answer this question, additional data layers were generated to simulate a future situation for each of the considered most dynamic risk factors. These datasets were subsequently used to generate future *S. mansoni* transmission risk areas. The first data layer generated represented the anticipated 2050 map of areas used for rice cultivation. This data layer was generated on the basis of the irrigation master plan^22^ and agriculture statistics and GIS data (unpublished). The second data set that was generated represented the anticipated spatial extent of future wetlands. Using the probability map of Rwandan wetlands as developed by Nyandwi et al.^12^, the extent of wetlands in 2050 was approximated using projected climatic changes. The projection of future climatic conditions for Rwanda was generated using the defined trend. Using more than 50 years of meteorological data, air temperature is following an increase of 0.35 °C per decade^23,24^ and Rainfall knew an increase of 4 mm per decade^25^. Other environmental (bio-physical and socio-economic) risk factors were assumed to remain constant.

### Statistical analysis and model inference

#### Zero-inflated Poisson model

**Figure 2:**
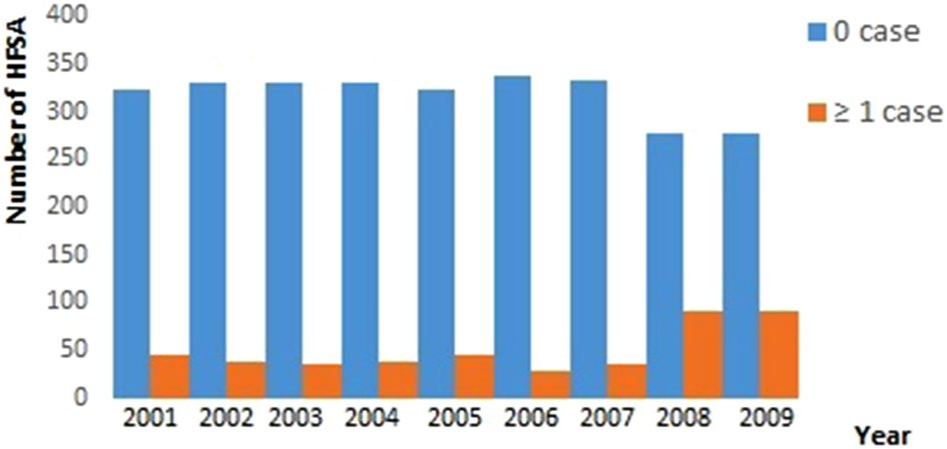
Risk factors maps. Categorical (from **a** to **f**) and continuous (from **g** to **i**) covariates.

Let *Y*_*it*_ be a random variable of *S. mansoni* outcomes with realizations *y*_*it*_ for *i* = 1, …, 367 HFSAs for *t* = 1, …, 9 years. It is assumed that the data are generated by a Poisson distribution. Initial exploratory analyses indicate the presence of numerous zeros with Pr(*y* = 0) = 0.29 (see Fig. 2). The number of zeros and the heterogeneous distribution of the positive counts imposes competing influences when the standard Poisson model is applied. Alternatively, we use zero-inflated Poisson likelihood to fit a three stage Bayesian hierarchical spatiotemporal model for *S. mansoni* risk. In this context, we model *y*_*it*_ as a mixture of Poisson distribution and a point mass at 0. Thus, when there are no cases, i.e. *y*_*it*_ = 0, we assume such zero counts are generated from a Poisson distribution with probability 1 − Φ_*it*_, and as ***sampling zeros*** with probability Φ_*it*_. Formally, this can be expressed as

$$ \Pr \left( {Y_{it} = y_{it} {{|\Phi }}_{it} ,r_{it} } \right) = \left\{ \begin{array}{ll} {{\Phi }_{it} + \left( {1 - {\Phi }_{it} } \right)\exp \left( { - r_{it} p_{it} } \right) } &\quad {{\text{for}} y = 0 } \\ {\left( {1 - {\Phi }_{it} } \right)\frac{{\exp \left( { - r_{it} p_{it} } \right)\left( {r_{it} p_{it} } \right)^{{y_{it} }} }}{{y_{it} !}}} &\quad {{\text{for}} y = 1,2,3, \ldots , } \\ \end{array} \right. $$

where *r*_*it*_ and *p*_*it*_ are the risk and population at the location *i*, respectively. Here, the zeros are a mixture of two distributions, the binary and the Poisson distribution that includes zeros. This distribution expresses the idea that an observation can be zero even though the disease is present. This is appropriate when there are detectability/diagnostic problems and/or deficiencies in surveillance systems, and the reluctance of infected individual to seek medical attention. We specified an alternative model assuming that only structural zeros are present and interpret the zeros as arising from only the binary distribution. In that case, we have the expression

$$ \Pr \left( {Y_{it} = y_{it} {|\Phi }}_{it} ,r_{it}  \right) = \left\{ \begin{array}{ll} {{\Phi }_{it} } &\quad {{\text{for}} y = 0 } \\ {\left( {1 - {\Phi }_{it} } \right)\frac{{\exp \left( { - r_{it} p_{it} } \right)\left( {r_{it} p_{it} } \right)^{{y_{it} }} }}{{y_{it} !}}} &\quad {{\text{for}} y = 1,2,3, \ldots , } \\ \end{array} \right. $$

At the process stage, our interest is to model the risk of infection *r*_*it*_ as a latent random field conditional on *y*_*it*_ containing either sampling or structural zeros. Thus

$$ {\text{log}} r_{it} = \beta_{0} + \sum f_{p} \left( {x_{ip} } \right) + \sum z_{itk}^{T} \gamma_{tk} + u_{i}^{CAR} + v_{i}^{iid} + t_{t}^{RW} + \varepsilon_{it} $$

where *β*_0_ is the intercept, *f*_*p*_ are appropriate smooth functions of *p* = 1, …, *P* continuous covariates *x*_*ip*_, and *z*_*itk*_ is a vector of *k* = 1, …, *K* categorical covariates with associated parameters *γ*_*tk*_. We specified $u_{i}^{CAR} \sim ICAR\left( {w,\sigma_{u}^{2} } \right)$ as intrinsic conditional autoregressive (ICAR) process with variance $\sigma_{u}^{2}$ and the random intercepts $v_{i}^{iid} \sim N\left( {0,\sigma_{v}^{2} } \right)$ as zero-mean Gaussian process with variance $\sigma_{v}^{2}$, Here the weight matrix *w* represent the spatial neighborhood structure. By convention, and as applied in most studies, two HFSAs are assumed neighbors if they share a common boundary. Thus, we defined neighborhoods as adjacent HFSAs with simple binary adjacency weights, i.e., *w*_*ij*_ = 1 if areas *i* and *j* share a common boundary and *w*_*ij*_ = 0 otherwise. For the continuous covariate *x*_*ip*_ of *M* equally spaced knots $x_{p}^{1} < x_{p}^{2} < \cdots x_{p}^{M}$, we specified the nonlinear function $f_{p} \left( {x_{ip} } \right) = \xi_{pm} ,m = 1, \ldots ,M$, with second-order random walk priors $\xi_{pm} \sim N\left( {2\xi_{p,m - 1} - \xi_{p,m - 2} ,\sigma_{p\xi }^{2} } \right)$ and non-informative priors for $p\left( {\xi_{1} } \right) \propto 1,p\left( {\xi_{2} } \right) \propto 1$ and $p\left( {\xi_{m > 2} } \right)\sim N\left( {0,0.001} \right)$. Here, the variance parameters $\sigma_{p\xi }^{2}$ control the amount of smoothing. Also for the temporal trend, we write $f\left( t \right) = \rho_{m}$ and specify second-order random walk prior $\rho_{q} \sim N\left( {2\rho_{q - 1} - \rho_{q - 2} ,\sigma_{\rho }^{2} } \right)$ and non-informative priors $p\left( {\rho_{1} } \right) \propto 1,p\left( {\rho_{2} } \right) \propto 1$ and $p\left( {\rho_{q > 2} } \right)\sim N\left( {0,0.001} \right)$. Lastly, we specified $\varepsilon_{it} \sim N\left( {0,\sigma_{\varepsilon }^{2} } \right)$ as zero-mean random space–time interaction effects. Let ${\varvec{\xi}} = \left\{ {\xi_{pm} } \right\}$ be a vector of *PM* parameters, ${\varvec{\rho}} = \left\{ {\rho_{q} } \right\}$ be a vector of *Q* temporal trend parameters, ${\varvec{\gamma}} = \{ \gamma_{tk} \}$ be a vector of *K* categorical parameters, and ${\varvec{\varepsilon}} = \{ \varepsilon_{it} \}$ be a vector of 3303 space–time parameters. The full Gaussian latent field is then ${{\varvec{\uppsi}}}_{1} = \left\{ {\beta_{0} ,{\varvec{\xi}},{\varvec{\gamma}},{\varvec{\rho}},{\varvec{u}},{\varvec{v}},{\varvec{\varepsilon}}} \right\}$.

At the third stage of the Bayesian hierarchical model, we treat the precision/variance and the fixed parameters as unknown and assign prior distribution for their joint density. For the intercepts parameters, we specified $p\left( {\beta_{0} } \right) \propto 1,p\left( {\xi_{1} } \right) \propto 1,p\left( {\xi_{2} } \right) \propto 1,p\left( {\rho_{1} } \right) \propto 1,p\left( {\rho_{2} } \right) \propto 1$. For the fixed effects, we specified non-informative Gaussian priors $p\left( {\xi_{m > 2} } \right)\sim N\left( {0,0.001} \right),p\left( {\rho_{q > 2} } \right)\sim N\left( {0,0.001} \right),p\left( \gamma \right)\sim N\left( {0,0.001} \right)$. For the precision parameters,$\tau_{j} = 1/\sigma_{j}^{2}$ we assumed $\tau_{j} \sim logGamma\left( {1,0.005} \right),j = \xi ,\rho ,u,v,\varepsilon$, as prior distributions. Following the Bayesian paradigm, we aim to determination the posterior distribution of the unknown parameters based on their prior distribution. Let ${{\varvec{\uppsi}}}_{2} = \left\{ {\tau_{\xi } ,\tau_{\rho } ,\tau_{u} ,\tau_{v} ,\tau_{\varepsilon } } \right\}$ be a vector of all unknown variance parameters, one can then simulate samples from the posterior density $p({\uppsi }_{1} ,{\uppsi }_{2} |y) \propto p(y|{\uppsi }_{1} ,{\uppsi }_{2} )p({\uppsi }_{1} |{\uppsi }_{2} )p\left( {{\uppsi }_{2} } \right)$ using Markov Chain Monte Carlo (MCMC) simulation. However, in this study, we generated the samples from the posterior distribution using the Integrated Nested Laplace Approximation (INLA)^25^. INLA is an emerging alternative to the MCMC that provides fast and accurate estimates of the posterior marginal through Laplace approximation, a deterministic algorithm proposed by Rue and Martino^25^. Details about this approach and its applications can be found elsewhere^25–27^.

#### Model implementation and performance evaluation

We implemented two models, model 1 and model 2 in the R-INLA package and discussed their statistical and substantive grounds. We specified the model with Eq. (1) as model 1 and the model with Eq. (2) as model 2. The full model was expressed as

$$ \begin{aligned} \log r_{it} & = \beta_{0} + f_{Wet} \left( {Wet} \right) + f_{Rain} \left( {Rain} \right) + f_{Temp} \left( {Temp} \right) + Rice^{T} \gamma_{Rice} + d_{Rice}^{T} \gamma_{{d_{Rice} }} \\ & \quad + d_{Water}^{T} \gamma_{{d_{Water} }} + u_{i}^{CAR} + v_{i}^{iid} + t_{t}^{RW} + \varepsilon_{it} \\ \end{aligned}. $$

We compared the predictive perfornces of models 1 and 2 using the deviance information criterion (*DIC*) suggested by Spiegelhalter et al.^28^. The $DIC = \overline{D}\left( \theta \right) + p_{D}$ is a two-term composite parameter of which the posterior mean deviance $\overline{D}\left( \theta \right)$ measures the fit to the data, whiles the effective number of parameters *p*_*D*_ measures the model complexity. In this regard, the smaller the *DIC* value the better the model fit and predictive performance.

Also, the relationships between observed and predicted relative risks were compared. A quasi-validation was also done by plotting observed and predicted relative risks during the study period (2001–2009).

### Ethical statement

This study used routinely registered aggregated data, not clinical samples, and stored from Rwanda Health Information and Management System (RHIMS). The accessibility of the dataset for this study was guaranteed by an agreement signed between the researchers and Rwanda Biomedical Centre (RBC).

## Results

**Figure 3:**
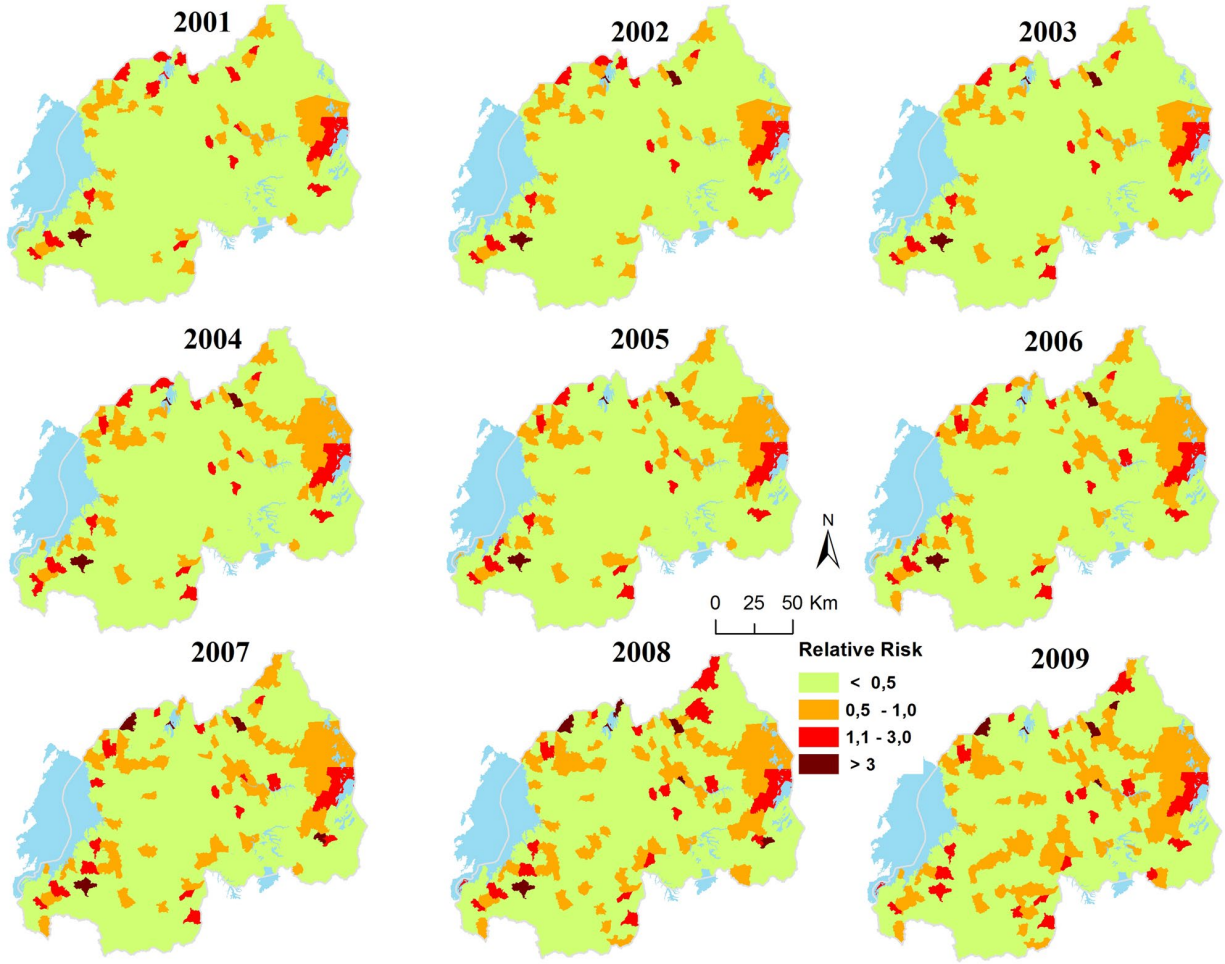
The annual relative risk of *S. mansoni* in Rwanda from 2001 to 2009.

Figure 3 displays the modeled endemic areas of *S. mansoni* at HFSA level. The annual risk *r*_*it*_ (RR) of *S. mansoni* depicts the changes in the period 2001 to 2009. The spatial pattern of transmission risk is rather dynamic across the country during the study period. Overall, 340 HFSAs have less than 1 case of schistosomiasis per 10,000 people for most of the years. Significant transmission risk (i.e.,*r*_*it*_ > 1) is predicted for about 37 HFSAs. Very significant risk (*r*_*it*_ > 3) is observed only in a very limited number of HFSAs (*n* = 3).

**Figure 4:**
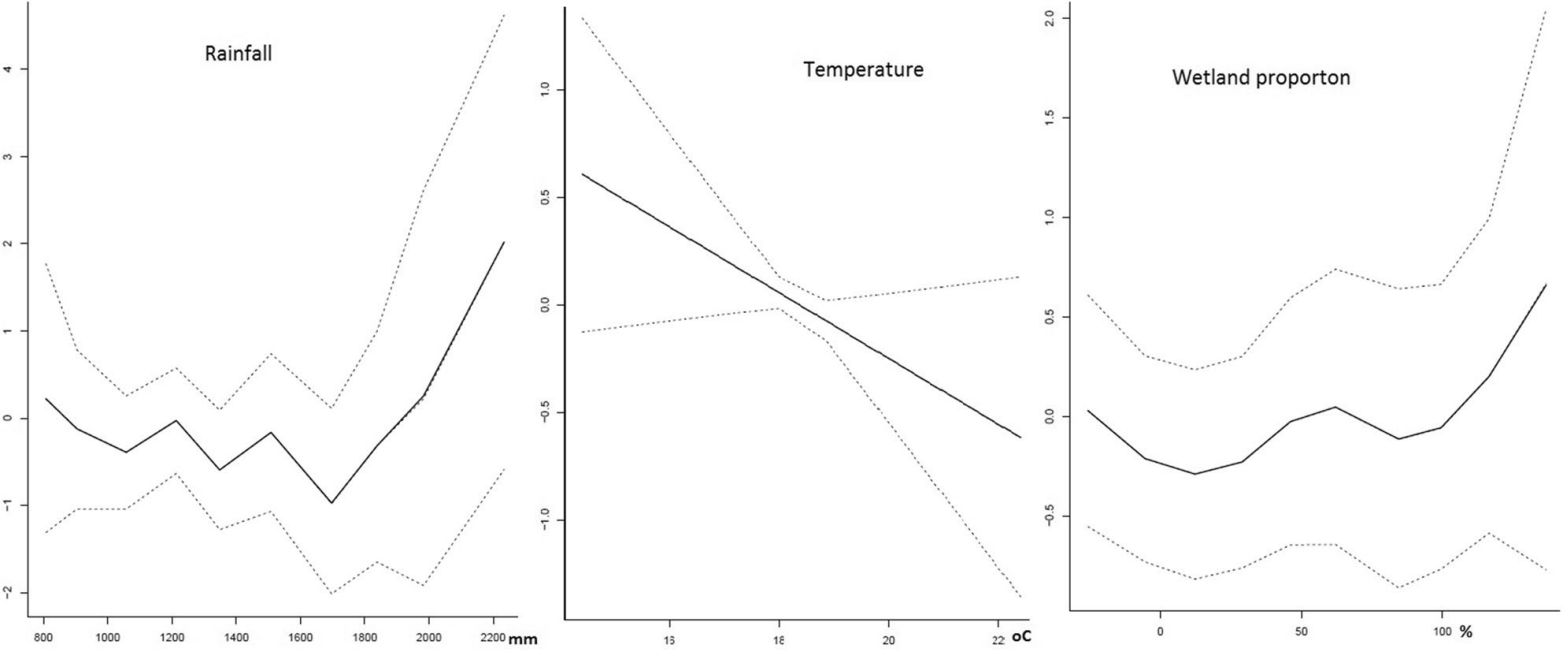
Model outputs from continuous variables.

As summarized in Table 1 and illustrated in Fig. 4, schistosomiasis infection risk is positively correlated with the presence of rice cropping, proximity to rice cropping areas, proximity to water bodies, wetland proportion, and rainfall at HFSA level. Furthermore, as illustrated by the curve of middle graph of Fig. 4, *S. mansoni* is negatively correlated with the air temperature. Model two performed best using the deviance information criterion (DIC). This model assumes that reported zeros cases for some HFSA were false zeros.

**Table 1:** Posterior estimates of the fixed effects of model parameters with ZIP distribution of the two models of Schistosomiasis risk in Rwanda, 2001 to 2009.

Parameters	Model 1			Model 2		
	Mean	Q_0.025_	Q_0.975_	Mean	Q_0.025_	Q_0.975_
*β*_0_	0.000134	0.000069	0.000263	0.0000785	0.00003	0.00019
*γ*_*Rice*_	1.397	0.882	2.2071	1.688	0.989	2.891
$\gamma_{{d_{Rice} }}$	1.289	0.765	2.166	1.292	0.669	2.485
$\gamma_{{d_{Water} }}$	1.322	0.828	2.098	1.498	0.837	2.693
*DIC*		4970.10			4547.91	
*p*_*D*_		296.45			336.84

**Figure 5:**
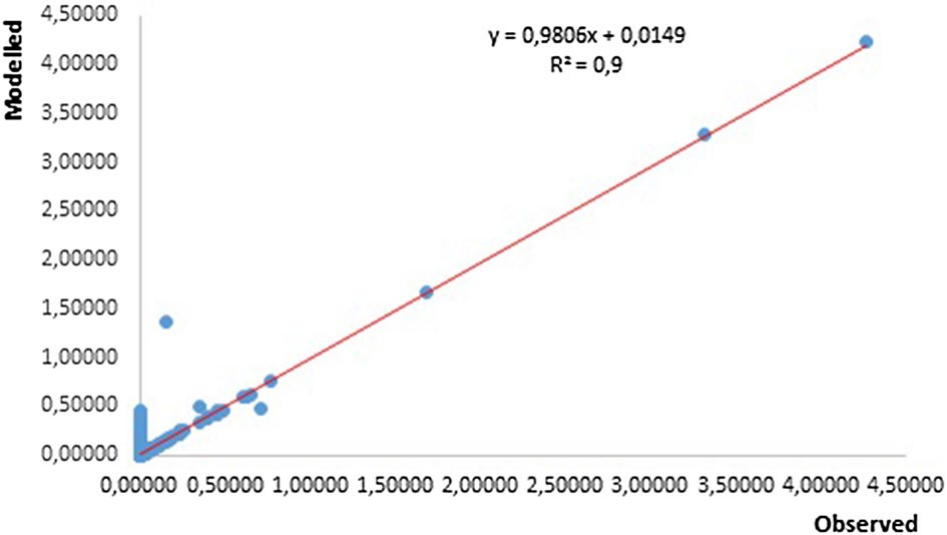
Scatter plot and trend line of Observed against modeled RR of schistosomiasis between 2001 and 2009.

In Fig. 5, shows a strong relationship between the observed and the modeled relative risk (RR), with a coefficient of determination of 0.9. The relationships increase the confidence in formulating the assumptions around the model outputs for forecasting the RR by 2050.

**Figure 6:**
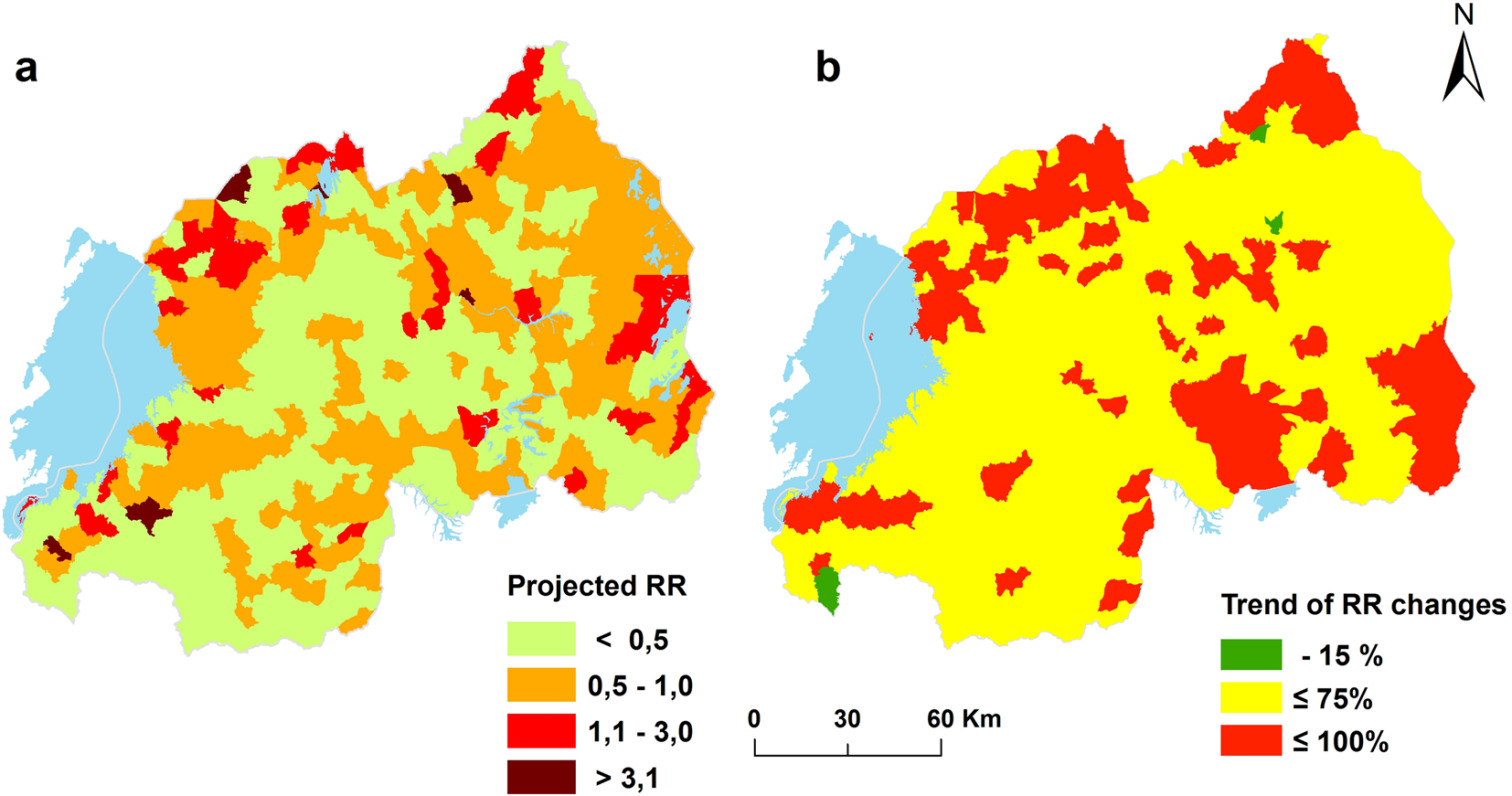
The future relative risk of schistosomiasis. Projected relative risk by 2050 (**a**) and change detected between 2009 and 2050 (**b**).

Figure 5 illustrates the probable spatial pattern of schistosomiasis considering the current temporal trend of the disease risk and projected trend of changes of associated risk factors by 2050. The number of endemic HFSAs (i.e*., r*_*it*_ > 1) will increase from 26 in 2009 to 46 in 2050. The number of HFSAs with a very significant risk of schistosomiasis (*r*_*it*_ > 3) will increase from 5 HFSAs in 2009 to nine in 2050. In general, by 2050 the relative risk will only decrease in three HFSAs, while there is a general increase of risk ranging from 5 to 98% as illustrated by Fig. 6b which depicts the change of schistosomiasis risk over time.

## Discussion

The zero-inflated Poisson regression model shows a very significant increase of relative risk of *S. mansoni* transmission. Furthermore, the change between the relative risk of 2009 and the forecasted risk of 2050 indicates an increasing trend. The number of HFSAs with significant risks will double by 2050 as illustrated by disease risk areas change the map in Fig. 6b. Although a lot has been achieved by the schistosomiasis control initiatives since 2008^4^, schistosomiasis risk areas remain, and vary across space and over time. This confirms the high level of uncertainty for modeling schistosomiasis risk pattern with linear regression approach. We also compared the two models, and the DIC results indicate that model 2, the model considering the existence of false zeros, is the best fitting model.

The increasing spatial variation of *S. mansoni* cases can be explained by the statistically significant association with water and rice related environmental factors, as summarized by the mean values in Table 1. The risk of *S. mansoni* transmission is 0.69, 0.29 and 0.50 (which means 69%, 29%, and 50% higher in a HFSA with rice cultivation, proximity to rice farms, and proximity to a water body respectively. The connection implies that *S. mansoni* infection will tend to be higher where peoples will concentrate around water development projects. Then, in the areas where probability of contact with contaminated water^29^ and/or to contaminate water is increased. *S. mansoni* infection will therefore predominantly affect the rural population whose water contact is linked to human ecology created around wetlands with intensified agriculture and areas around others water related development projects such as multi-purpose water dams^20,30,31^. Furthermore, climatic factors also add to risk because warmer and more humid conditions tend to stimulate several soil- and water-associated diseases and their host, snails in our case^32^. Thus, the different direction of correlation between schistosomiasis risk and air temperature is in line with the fact that warmer conditions increase evapotranspiration leading to dryer conditions and reducing the susceptibility of snails to become infected with Schistosoma^33^.

The identified trend for disease pattern with significantly associated non-fixed environmental factors was also maintained in forecasting the disease by 2050. Air temperature and total amount of rainfall data captured over the past 50 years^23^ were used to generate a projection for 2050 and their significant correlation with observed disease risk might have to be maintained in disease risk forecast. Likewise, the projection of a water body, wetlands, and rice cropping related factors was based on local realities. However, our results should be interpreted with caution because of our *ceteris paribus* assumption, all other factors remain constant over time. Available socio-economic data such as the proportion of households with improved sanitation, access to clean water and level of education were included in the initial model but did not show significant contribution and were therefore not retained in the model. Probably this is due to the coarse spatial scale of these data. In the study period a nationwide NTD control program was initiated and the impact of improved disease diagnostic and drug treatment reflected in reducing schistosomiasis incidence in many parts of the country^13^. But the same study has shown that *S. mansoni* persists in traditional endemic areas and also emerges in new areas. Surprisingly, the traditionally endemic area (Bugarama) in the extreme southwest of the country exhibits a drastically reduced risk forecast for 2050 (Fig. 5b). The value in time and space of forecasted risk should be used with some caution by public health professional and decision-makers in planning for disease control interventions^34^.

## Conclusion

We selected a Bayesian statistical model adequate for analyzing the spatiotemporal distribution of schistosomiasis infection risk using routine health records HFSAs with true as a well as false zeros. Structured additive models combining continuous and categorical variables revealed a spatiotemporal pattern of increasing schistosomiasis infection risk over time, significantly associated with presence of or proximity to water bodies and rice cultivation, the proportion of wetland cover, and total rainfall amount and air temperature. Furthermore, the forecasting results showed both persisting and emerging HFSAs with a high relative risk of schistosomiasis infection. The prediction and forecasting maps provide a valuable tool for monitoring schistosomiasis risk in Rwanda and can provide valuable inputs for planning of future disease control initiatives. According to the forecast for 2050 and following WHO guideline, three districts of the Eastern Province would be added to the list of endemic zones to benefit from Mass Drug Administration and other control programs, if there will be no public health measures accompanying expanding rice cropping.
